# Functional Aspects of PARP1 in DNA Repair and Transcription

**DOI:** 10.3390/biom2040524

**Published:** 2012-11-12

**Authors:** Hui Ling Ko, Ee Chee Ren

**Affiliations:** 1 Singapore Immunology Network, A*STAR, 8A Biomedical Grove, #03-06 Immunos, Singapore 138648, Singapore; Email: Ko_Huiling@immunol.a-star.edu.sg (H.K.); 2 Department of Microbiology, Yong Loo Lin School of Medicine, National University of Singapore, 5 Science Drive 2, MD4, Level 5, Singapore 117597, Singapore

**Keywords:** ADP-ribosylation, DNA repair, transcription, PARP inhibitors, cancer, inflammation, oncogenic virus

## Abstract

Poly (ADP-ribose) polymerase 1 (PARP1) is an ADP-ribosylating enzyme essential for initiating various forms of DNA repair. Inhibiting its enzyme activity with small molecules thus achieves synthetic lethality by preventing unwanted DNA repair in the treatment of cancers. Through enzyme-dependent chromatin remodeling and enzyme-independent motif recognition, PARP1 also plays important roles in regulating gene expression. Besides presenting current findings on how each process is individually controlled by PARP1, we shall discuss how transcription and DNA repair are so intricately linked that disturbance by PARP1 enzymatic inhibition, enzyme hyperactivation in diseases, and viral replication can favor one function while suppressing the other.

## 1. Introduction

Poly (ADP-ribose) polymerase 1 (PARP1) is well known as an ADP-ribosylating enzyme, which becomes activated upon binding to DNA single-strand and double-strand breaks (ssDB and dsDB respectively) [1,2,3,4,5,6]. The interaction is important for DNA repair, as auto-ribosylation is necessary to assemble and activate multiprotein complexes to carry out the process [6,7]. The critical role of PARP1 in DNA repair is reflected by its frequent upregulation in cancer [8,9], as well as the hypersensitivity of PARP1 null animals towards the mutagenic effects of DNA damaging agents [10]. Since PARP1 is involved in the repair of modified bases, ssDB and dsDB [7], blocking the ADP-ribosylation activity with small molecules, can achieve synthetic lethality with DNA damaging agents in the treatment of cancer [8,9,11,12,13,14,15,16,17]. Besides DNA repair, the importance of PARP1 as a transcriptional regulator is also well established. As an enzyme, PARP1 acts on chromatin remodeling complexes to control DNA accessibility for RNA polymerase [18,19,20,21,22]. PARP1 also functions as a transcription factor by binding an octamer motif in promoter elements to regulate gene expression [23,24,25,26,27,28,29,30]. Interestingly, despite the roles of PARP1 in DNA repair and transcription, little is known about how one process affects the other. In this review, we will summarize the roles of PARP1 in DNA repair and transcriptional regulation. Through the motif–PARP1 interaction, we will also discuss how transcription and DNA repair affect one another in normal cell functions and diseases states.

## 2. PARP1 Function and Regulation

**Figure 1:**
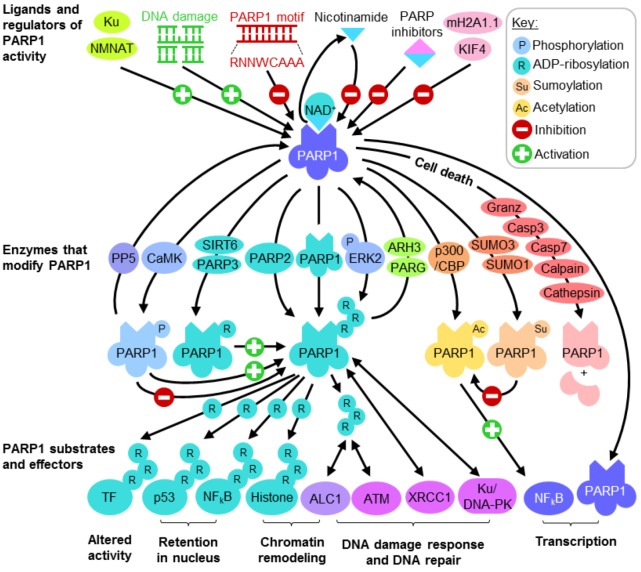
PARP1 function and regulation. Abbreviations: Granz: Granzyme; Casp:Caspase; TF: Transcription factor.

PARP1 is the first to be identified among a family of 17 proteins that cleaves NAD^+^ for the ADP-ribosylation of protein acceptors, generating nicotinamide as a by-product [16,31]. The large 113kDa nuclear protein usually has low intrinsic enzymatic activity [32] which may be significantly enhanced by binding both ssDB and dsDB via either of its *N*-terminal zinc fingers [33], bringing about conformational changes through its third zinc finger to increase catalytic activity at the *C*-terminal [3,34,35]. As large amounts of negative charges are conferred by adding extensive polymers of ADP-ribose (PAR), PARP1 modulates the activity of its substrates, including itself, to control several important cellular functions (Figure 1) such as DNA damage repair, transcriptional regulation and cell death [6,16,19,20,21,22,31,36,37,38,39,40,41,42]. However, PAR is short-lived and as soon as its purpose is served, it is rapidly degraded within minutes of synthesis by the exoglycosidic and endoglycosidic activities of poly (ADP-ribose) glycohydrase (PARG) or PAR hydrolase (ARH) [6].

### 2.1. Regulating PARP1 ADP-Ribosylation Activity

PARP1 enzymatic activation accounts for the bulk of cellular ADP-ribosylation reactions [43,44] and consumes large amounts of NAD^+^. The accumulation of PAR is a cytotoxic signal as targeted disruption of PARG is shown to be embryonically lethal in mice and associated with apoptotic cell death in blastocysts [45]. Not surprisingly, PARP1 enzymatic activity is thus regulated at several levels (Figure 1). Acting directly at the catalytic domain, the by-product nicotinamide exerts mild inhibitory effects on PARP1 ADP-ribosylation activity [8,9,14,15,17]. The molecular basis for interference with NAD^+^ binding is well studied, and often imitated or improved upon when designing high affinity small molecule inhibitors targeting PARP1 functions. The end product PAR also helps to limit NAD^+^ consumption by mildly inhibiting PARP1 and, when becoming highly branched, confers excessive negative charges for repulsion from DNA, switching off ADP-ribosylation. Interestingly, while the best characterized ligands for the PARP1 enzyme are ssDB and dsDB, PARP1 has a higher affinity for intact DNA and specifically recognizes the octamer motif “RNNWCAAA” found in various gene promoters [23]. This interaction suppresses PARP1 ADP-ribosylation activity and interferes with its enzyme-dependent functions.

Various posttranslational modifications are known to regulate PARP1 enzyme activity. PARP1 may be acted upon by other members of the PARP family such as PARP2, and mono-ADP-ribosylation by PARP3 has been shown to enhance PARP1 activity and automodification [46]. Other enzymes possessing ADP-ribosylating activity, such as SIRT6, also act on PARP1, promoting dsDB repair in oxidative stress [47]. Depending on which residue is modified on the large protein, PARP1 phosphorylation seems to exert differential effects on its ADP-ribosylation activity. In the presence and absence of damaged DNA, its catalytic activity may be significantly enhanced and maximized by action of phosphorylated ERK2 [48,49,50]. Similarly, phosphorylation by activated calcium-dependent protein kinase (CaMKII) during neuronal development activates PARP1 enzyme and promotes the nuclear export of its negative regulator KIF4 [51]. However, overexpression of protein phosphatase 5 (PP5) increases PARP1 enzymatic activity towards dsDB [52], suggesting that the kinases and PP5 act on different residues to bring about contrasting effects on PARP1 enzymatic activity. Intricate cross-talks between PARP1 acetylation and sumoylation have also been observed, where modification by SUMO1 and SUMO3 prevents p300-mediated acetylation of PARP1 [53]. Given the number and types of posttranslational modifications PARP1 is subjected to, it would not be surprising to find more of such cross-talks. 

Besides direct interference with catalysis and molecular switches by posttranslational modification, several proteins are known to bind and modulate PARP1 activity. Perhaps acting as a convenient source of substrate, nuclear NMNAT catalyzes the final step of NAD^+^ synthesis and associates with PAR to enhance PARP1 enzyme activity [54]. Other proteins regulating PARP1 activity include Ku [52], histone variant mH2A1.1 [55] and KIF4 [56]. Another important strategy often used in cell death pathways to control PARP1 activity is cleavage by proteases. In early apoptosis, PARP1 is one of the first substrates cleaved by caspase 3 and caspase 7 between the second and third zinc-binding domains [57,58], preventing DNA strand-break binding from inducing NAD^+^ catalysis [40,59,60]. In immune responses, cytotoxic lymphocytes and NK cells release of granzyme A and granzyme B that also target PARP1 [61]. In response to intracellular calcium perturbations, PARP1 may also be cleaved by calpain 1 [61,62]. Furthermore, PARP1 cleavage is also observed in autophagic and necrotic cell death pathways by action of various cathepsins [61,63]. The cleavage of PARP1 by a myriad of proteases at different loci seems to be a common mechanism in cell death, however, the significance of this is not yet fully understood. 

### 2.2. PARP1 Substrates and Effectors Play Important Roles in Transcription and DNA Damage Response

PARP1 is itself the major acceptor of PAR [43]. The extensive branching network of PAR on PARP1 acts as the cue to attract and assist in assembling multiprotein complexes involved in chromatin remodeling, DNA repair and damage checkpoint signaling (Figure 1) [50,64,65,66,67,68,69]. Histones such as H1 and H2B are important substrates of PARP1 [22,65,70,71], which, when displaced by ADP-ribosylation, enables enhanced accessibility of large protein complexes assembled during DNA repair and transcription. After strand-break dependent activation, DNA repair scaffold proteins such as XRCC1 may be directly recruited by automodified PARP1 [72], while PAR provides the localization signal for directing the nucleosome repositioning enzyme ALC1 (Amplified in Liver Cancer 1) [73] in response to DNA damage. PAR also recruits the DNA damage checkpoint protein ATM (Ataxia Telangiectasia Mutated) [74], activating the signaling cascade for DNA damage and cell cycle arrest. Besides recruiting and activating nuclear complexes, PARP1 also exerts its effects by directly modifying protein activity and localization. Transcription factors and transcription coregulators such as SP1, Oct-1 and hnRNP K (heterogeneous nuclear ribonucleoprotein K) are known targets of PARP1, when ADP-ribosylated are repelled from DNA hence resulting in altered transcript expression profiles [31,75,76,77]. The bulky posttranslational modification prevents the association of transcription factors p53 and NF-κB to nuclear export factors such as Crm1, enabling nuclear retention [78,79]. Thus, through altering transcription factor function and localization, as well as remodeling chromatin structure and recruiting DNA processing complexes, PARP1 plays pivotal roles in both transcriptional regulation and DNA damage response.

## 3. PARP1 ADP-Ribosylation Activity Is Important for Mediating DNA Repair

Though not directly involved in any of the processes *per se*, PARP1 initiates and modulates multiple DNA repair pathways (Table 1) and is thus important for maintaining genomic integrity. Indeed, PARP1 knockout mice are highly susceptible to DNA damaging agents such as γ-irradiation and DNA alkylating agents, accounting for DNA strand break accumulation, increased sister chromatid exchange, and high genomic instability in them [80]. While these mice are viable and phenotypically normal [80], PARP1 knockout mice haploinsufficient for DNA repair enzymes such as Ku80 have increased spontaneous mutations and present higher liver and brain tumor incidence with age [81,82]. Female PARP1 knockout mice also develop mammary carcinomas with age, which is accelerated with the loss of p53 [83]. The importance of PARP1 for DNA repair is further demonstrated by embryonic lethality in knockout mice models doubly-deficient for PARP1 and DNA repair proteins Ku80 [82], BRCA1 [84], ATM [38] or DNA polymerase β [85]. The requirement for PARP1 in DNA damage repair is dependent on its ADP-ribosylation activity, as all male rats fed on a diet to deprive liver NAD^+^ spontaneously developed hepatocellular carcinoma with age [86].

**Table 1:** Involvement of PARP1 and PAR in DNA repair pathways.

DNA Repair Mechanism	PARP1 Function	References
Base excision repair (BER)	Binds AP site	[87]
	Auto-modified PARP1 recruits BER complex	[88]
Nucleotide excision repair (NER)	ADP-ribosylates XPA	[89,90]
Mismatch repair (MMR)	ADP-ribosylates MSH6	[89,90]
Single-strand break repair (SSBR)	Auto-modified PARP1 recruits BER complex	[89,91,92]
Double-strand break repair by nonhomologous end joining (NHEJ)	Ku enhances PARP1 ADP-ribosylation activity	[89,90]
	ADP-ribosylates and activates DNA-PKcs	
Double-strand break repair by homologous recombination (HR)	Auto-modified PARP1 recruits Mre11	[66]
	PAR activates ATM signalling	[74]

### 3.1. PARP1 in the Repair of Modified DNA

**Figure 2:**
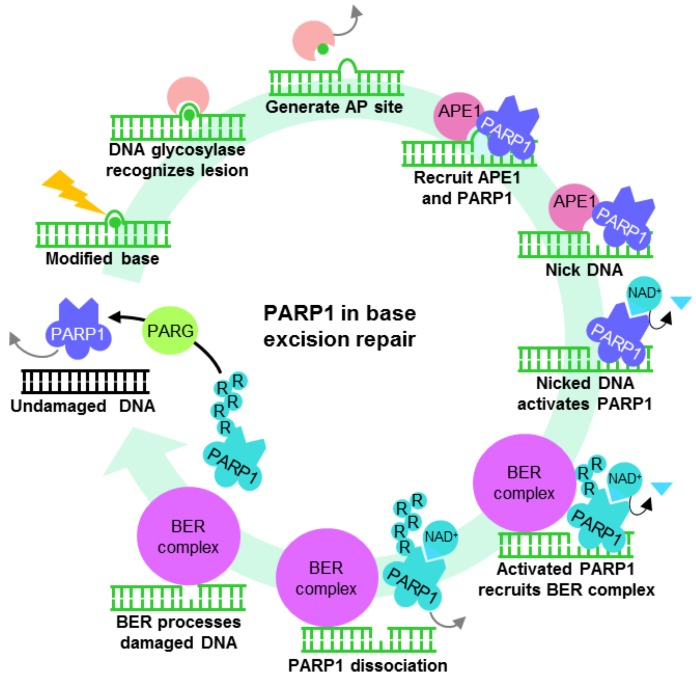
The role of PARP1 in base excision repair (BER). PARP1 may also mediate DNA single-strand break repair by recruiting the BER complex.

Several DNA repair pathways are in place to tackle a variety of genotoxic lesions. These pathways are activated depending on the type of DNA insult and phase within the cell cycle. Minor damage to bases such as methylation may be chemically reversed by specific glycosylases such as MGMT in an energy inefficient “suicide” reaction, restoring the base but rendering the enzyme unusable for subsequent reactions. To overcome this, base excision repair (BER) complexes targeting nonbulky modified bases may be recruited by PARP1 (Figure 2). Base modification by methylation, deamination and oxidation are recognized and removed by specific DNA glycosylases, generating an apurinic/apyrimidinic site (AP site) which, through a mechanism that is not completely understood, recruits PARP1 and APE1 [87]. APE1 removes the deoxyribose phosphate backbone at the site of lesion, generating nicked DNA which significantly enhances PARP1 ADP-ribosylation activity. The highly charged PAR produced keeps the DNA structure open [1] while various components of the BER complex are recruited. These components include the scaffold protein XRCC1 (X-ray Repair Cross-Complementing Protein 1), the DNA end-processing kinase/phosphatase PNK (Bifunctional polynucleotide phosphatase/kinase), the gap-filling polymerase DNA polymerase β and DNA ligase III [91,92] (Figure 2). By the time the BER complex is assembled, PARP1 accumulates enough negative charges for repulsion from the DNA lesion, enabling the BER and subsequent ligation to restore DNA. 

Large adducts that distort DNA structure such as thymine dimers formed by UV irradiation require nucleotide excision repair (NER) for resolution [91,93]. In transcriptionally quiescent cells, global genomic NER (GG-NER) is initiated by the recruitment of XPC/HHRAD23B complex whereas in cells undergoing active transcription, the stalled RNA polymerase II is displaced with the aid of CSA and CSB proteins in transcription coupled NER (TC-NER). The two sub-pathways converge with the unwinding of DNA at the site of damage by transcription factor IIH, and the recruitment of DNA lesion recognition factors XPA and RPA. The endonucleases XPG and ERCC1/XPF then cut one strand of the unwound DNA at either ends of the damage to produce a 23–30 nt fragment containing the DNA lesion. While the resultant gap in DNA is not known to bind PARP1, XPA interestingly has been shown to be associated with PAR [90,91] (Table 1). Prior to DNA ligation the gap is repaired by DNA polymerase δ or ε, together with factors including PCNA, RPA and RFC.

When faced with singly or doubly mismatched bases and small loops arising from insertions or deletions, lesion recognition by MUTSα (MSH2-MHS6 heterodimer) initiates DNA mismatch repair (MMR) [92] and the recruitment of MUTLα (PMS2-MLH1 heterodimer). Conformation changes allow MUTSα-MUTLα to move along and nick DNA near the mismatch, enabling EXO1 (Exonuclease 1) to cleave and remove the damaged section of DNA. It is unclear whether PARP1 is activated at the site of DNA strand break, but MSH6 has been shown in independent studies [89,90] to be an acceptor of PAR (Table 1). Subsequently, gap filling by DNA polymerase δ or ε followed by ligation restores DNA.

### 3.2. PARP1 in the Repair of DNA Strand Breaks

DNA strand breaks may be directly induced by γ-irradiation or X-rays, as well as drugs such as bleomycin [51]. Under physiological conditions, they can be purposefully induced to enhance genetic diversity for meiotic recombination and antibody class switching [94]. Stalled replication forks may also require dsDB for resolution [66]. Single-strand DNA breaks are readily repaired by the BER complex, as PARP1 readily recognizing nicked DNA to organize its recruitment. The repair of dsDB is trickier as the involvement of both DNA strands prevents the use of neither as template. Thus, two pathways have evolved to repair such DNA lesions—nonhomologous end joining (NHEJ) and homologous recombination (HR). Even though their mechanistic details have not been fully elucidated, recent data indicates that PARP1 is not only involved in them (Table 1) but possibly also controls the choice of pathway utilized [95,96,97,98]. 

HR repairs DNA with high fidelity, using either homologous chromosomes in G1 phase or the sister chromatid after DNA replication as the blueprint for repair [99]. Following end recognition, the MRN complex comprising Mre11 nuclease, Rad50 and Nbs1 is recruited along with CtIP complex bearing BRCA1 to resect DNA, generating 3’ single-stranded overhangs which are stabilized by RPA1. PARP1 may contribute to this process at stalled replication forks by binding short single-stranded overhangs and recruiting Mre11 [66]. MRN also activates ATM signaling, initiating the DNA damage response including cell cycle arrest. BRCA2 then mediates the exchange of RPA1 for RAD5, and directs the presynaptic filament in its search for homologous DNA template. Strand invasion and exchange ensues, allowing DNA polymerase to extend the 3’ end of the invading strand. Repaired heteroduplexed DNA is generated after DNA ligase I joins the DNA ends and the resultant holiday junctions resolved by resolvases. When the damaged DNA may not be rapidly repaired, ssDNA-RPA1 may activate ATR signaling via DNA resection to sustain the DNA damage response [100,101,102,103,104]. This involves RPA1 interaction with ATRIP, RFC-mediated loading of the 9-1-1 clamp, and subsequent recruitment of TOPBP1 (topoisomerase binding protein 1) necessary for ATR activation. 

NHEJ is an error-prone mechanism that ligates DNA ends together, often occurring in G1 phase when a suitable repair template is not available [105]. The ring-structured Ku heterodimer comprising Ku70/Ku80 slips and binds onto the broken ends of DNA, recruiting other factors such as DNA-PK (DNA-dependent protein kinase), XRCC4 and DNA ligase IV. DNA strand-break binding activates the catalytic subunit of DNA-PK (DNA-PKcs), initiating DNA damage signaling cascade by autophosphorylating substrates including ATM, p53 and itself, while DNA end-processing enzymes such as Artemis prepares the damaged DNA for ligation. Recent data points to the recruitment of MRN for end processing, as well. As V(D)J recombination for antibody class switching utilizes the NHEJ machinery, the unusual antibody profiles of PARP1 knockout mice point to its involvement in the process [94]. Instead of directly binding dsDB, PARP1 ADP-ribosylation activity is strongly enhanced by interaction with Ku, forming a functional complex with DNA-PK [52]. The kinase activity of DNA-PK is significantly increased by ADP-ribosylation [106]. However, the precise role of PARP1 in NHEJ is still unclear.

### 3.3. Uncontrolled PARP1 ADP-Ribosylation Activity during DNA Repair Results in Cell Death

When DNA damage is minimal, the recruitment of PARP1 to sites of DNA lesions activates the DNA damage response. Depending on the type of lesion encountered, signaling mediated by molecules such as p53 and ATM or ATR to promote cell cycle arrest, buying time for DNA repair enzymes to work. However, when DNA damage is extensive and irreparable, PARP1 is rapidly cleaved within minutes of DNA damage by effector caspases [107,108], presumably to prevent futile DNA repair when DNA is eventually cleaved later in the process. The dissociated *N*-terminal fragment is believed to remain bound to DNA strand breaks while the *C*-terminal is rapidly shuttled out of the nucleus. While the reason for PARP1 cleavage is not clear, their different subcellular localizations is thought to conserve energy required for apoptosis by preventing unnecessary NAD^+^ consumption. In support of this, energy failure by making large amounts of PAR when DNA is extensively damaged results in cell death by necrosis instead [8,16,40,41,59,109]. PAR can also mediate PARP1-dependent cell death (parthanatos) through the release of Apoptosis-inducing factor (AIF) from mitochondria in a caspase-independent manner [42,110]. 

## 4. PARP1 as a Transcriptional Regulator Controlling Expression of DNA Damage Response Genes

**Figure 3:**
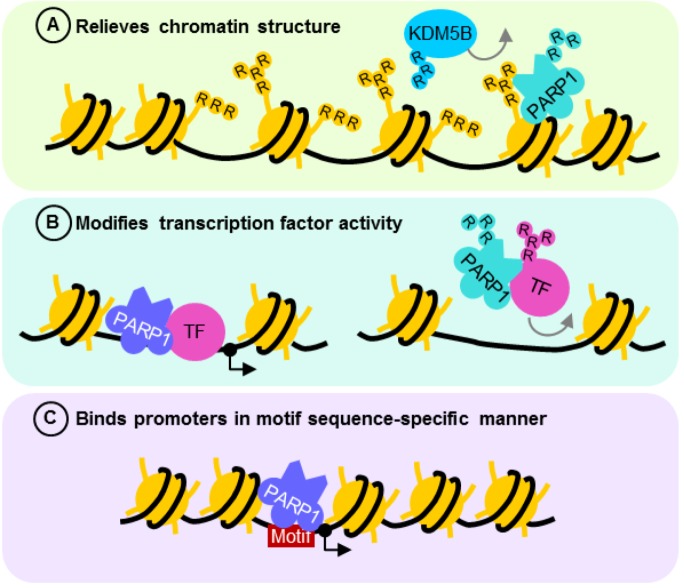
The role of PARP1 in transcriptional regulation. (A) PARP1 relieves and maintains an “open” chromatin structure by ADP-ribosylation of histones or preventing the action of histone demethylase KDM5B. (B) PARP1 forms functional complexes with transcription factors such as NFκB, altering their activity depending on its state of posttranslational modification. This interaction need not activate or require PARP1 enzyme activity, although when stimulated, ADP-ribosylation usually reduces the affinity of the complex for DNA cis elements. The effect of PARP1 on transcription in both cases is dependent on the type of binding partner and nature of promoter element recognized. (C) PARP1 acts as a transcription activator or repressor by binding its recognition motif. Grey arrows are repulsion from DNA or chromatin. R—ADP-ribosylation; TF—transcription factor.

PARP1 is an important regulator of transcription, as can be seen at PAR-rich *Drosophila* chromosomal “puffs” undergoing active transcription [111]. The importance of PARP1 in controlling transcription was further supported by global alterations in gene expression [112,113,114], most notably of those involved in cell cycle, DNA repair and metabolism. Transcriptional regulation by PARP1 involves both ADP-ribosylation-dependent and independent mechanisms (Figure 3). Furthermore, PARP1 exerts its effects on transcription both in a DNA sequence-dependent and independent manner, through motif recognition at specific gene promoters [24,25,26,27,30,115,116,117,118,119,120,121,122,123] and chromatin remodeling [20,22], respectively. 

### 4.1. PARP1 ADP-Ribosylation Activity Controls Transcription States

The “opening” of chromatin for active transcription often requires PARP1 ADP-ribosylation activity (Figure 3A) [18,22]. In the absence of NAD^+^, minimally automodified PARP1 acts as a transcriptional repressor by bridging neighboring nucleosomes to compact chromatin [18]. However, when enzymatically activated, the extensive negative charges conferred by automodified PARP1 loosens chromatin structure, thereby enabling transcription factors to bind. Upon recognition of its response element, transcription activators such as estrogen receptor α recruit a complex containing topoisomerase II-β and PARP1 [65]. While the topoisomerase resolves DNA secondary structures by creating transient dsDB, the DNA lesion activates PARP1 to ADP-ribosylate histones H1 and H2B. The negative charges on ADP-ribosylated histones repel DNA, loosening chromatin for increased DNA accessibility to the transcriptional machinery [18,22]. PARP1 is thus found in place of histone H1 in most transcriptionally active genes [21,69,70,113]. Modified histone H1 may also then be exchanged for histone H1-HMGB (histone H1 high mobility group B) favorable for transcription [65]. To maintain chromatin in its transcriptionally active state, PARP1 also prevents the histone demethylase KDM5B from approaching trimethylated histone H3K4 by repelling it from DNA through ADP-ribosylation [69]. The action of PARP1 on chromatin and chromatin remodeling complexes thus enables RNA polymerase II to load readily onto transcriptionally active promoter regions [69]. 

By modulating the affinity of transcription factors for their response elements and interacting partners, ADP-ribosylation acts as a molecular switch to control transcription [76,124,125,126,127] (Figure 3B). Through direct protein–protein interaction, PARP1 behaves like a coactivator or corepressor by forming a stable complex with transcription factors and its associated DNA cis element. Upon stimulation, the active PARP1 enzyme acts on its binding partner and results in complex dissociation from DNA. Transcription factors whose function is regulated by PARP1 in this manner include Oct-1 [76], SP1 [124], PPARγ [125], Smad3/Smad4 [126] and Sox2 [127], accounting for the dysregulation of multiple genes, hence the perturbation of several cellular processes in the absence of PARP1. For instance, transcription initiated by functional interaction between Smad3/Smad4 and PARP1 is disrupted when TGFβ1 signaling activates the PARP1 enzyme, attenuating Smad-dependent gene transcription for epithelial-mesenchymal transition [126]. Likewise, FGF/ERK signaling regulating embryonic stem cell differentiation relieves Sox2 interaction with its DNA responsive element by enhancing PARP1-dependent ADP-ribosylation [127]. 

PARP1 can also exert its effects as an inactive enzyme by directly interacting with and altering transcription factor function (Figure 3B). While ADP-ribosylated NFκB is retained in the nucleus [78], PARP1 controls its transcriptional activity [53,64,68,128,129] in an enzyme-independent manner. This is seen when acetylated PARP1 association with NFκB results in transcription of its downstream targets [64]. Sumoylation of PARP1, however, prevents p300/CBP from acetylating PARP1, hence the loss of coactivator function [53]. Surprisingly, in response to inflammatory stimulation by lipopolysaccharide, PARP1 loses its repressive function on NFκB at different sets of gene promoters when cleaved by inflammasome-dependent caspase 7, enabling transcription of pro-inflammatory genes [130]. Taken together, PARP1 is shown to be important for regulating transcription at multiple levels—from macroregulation of chromatin structure to the complex fine-tuning of gene expression which is dependent on its state of posttranslational modification. 

### 4.2. PARP1 is a Motif-Dependent Transcription Factor

Evidence for the sequence requirement of PARP1 transcription factor function (Figure 3C) came from studies of single nucleotide polymorphisms (SNP) within promoters that alter the affinity of PARP1 for undamaged DNA. For example, a single C➔T SNP within the IFNAR1 (Interferon-α/β receptor 1) promoter reduces PARP1-dependent transcription hence confers increased susceptibility towards chronic infection from the hepatitis B virus (HBV) [27]. Likewise, increased affinity of PARP1 for the SMARCB1 promoter by a single G➔T SNP enhanced the SWI/SNF chromatin remodeling complex transcript and protein expression [26]. In agreement with phenotypes from promoter SNP variant analysis, single base substitutions within the HBV PARP1 binding motif was sufficient to abrogate transcriptional activation at the viral core promoter [23]. The effect of single base substitutions on promoter transcriptional activity concurred with the ability of PARP1 to bind the mutant DNA sequence [24], indicating that the motif through which PARP1 exerts transcriptional effects is “RNNWCAAA,” where “R” is “A” or “G,” and “W” is “A” or “T,” and “N” may be any nucleotide. Motif recognition is heavily reliant on the 3’ half of the sequence, especially at nucleotide positions 5 and 6, as their mutation abrogated transcription and PARP1 binding. This recognition motif may be readily identified in other gene promoters whose activities are also regulated by PARP1, including immune regulators interferon-γ [119] and IL-10 [123], response elements of viruses such as the human T-cell leukemia virus (HTLV) [24], as well as BRCA2 [120]. Since BRCA2 is crucial for dsDB repair by HR, the finding that its expression is regulated by PARP1 suggests another means through which PARP1 controls DNA repair. Indeed, promoters of DNA repair genes often contain PARP1 binding motifs within 3kb upstream of the transcription start site (Table 2), many of which have important functions in HR. 

**Table 2:** Genes involved in DNA repair that possess the PARP1 binding motif.

DNA repair mechanism	Gene	Gene function [References]	PARP1 motif
**Double-strand break repair by homologous recombination (HR)**	BRCA1	E3 ubiquitin ligase with multiple roles including controlling DNA damage signaling [131]	GAAACAAA
	BRCA2^#^	Mediates recombination [132,133]	GGTACAAA
	BRIP1	Interacts with BRCA1 [131]	AGTTCAAA
			GAGTCAAA
	OBFC2B	SOSS complex component; ATM signaling [134]	GCGACAAA
	SSBIP1	SOSS complex component; ATM signaling [134]	GAGACAAA
	TOPBP1	Stalled replication forks; ATR signaling [135]	ATTTCAAA
			ATTTCAAA
	NSMCE2	E3 SUMO ligase of SMC5-SMC6 complex [136]	GGATCAAA
	SLX1B	SLX1-SLX4 resolvase catalytic subunit [137,138]	AGGACAAA
	DMC1	Meiosis-specific recombinase; Interacts with BRCA2 [132,139]	AGAACAAA
**Base excision repair (BER)**	NEIL3	DNA glycosylase [140]	AGCTCAAA
			AACACAAA
	MBD4^	DNA glycosylase specific for G:T or G:U mismatches within CpG islands [141,142]	ACAACAAA
**Nucleotide excision repair (NER)**	CETN2	Component of XPC complex [143]	GAGACAAA
**Mismatch repair (MMR)**	MSH6	Component of MMR [91]	GGGTCAAA
**Direct base reversal**	ALKBH3	Oxidative demethylation of alkylated DNA [144,145]	GCCACAAA
**Interstrand crosslink repair (ICL)**	FANCG	Component of FA core complex [146]	ACTACAAA
**DNA repair accessory proteins**	RPA1	Stabilize single-strand DNA intermediates	GTGACAAA
**DNA polymerases**	POLA2	Subunit of primase complex	GCTACAAA
	POLD3	DNA polymerase δ subunit	ACTTCAAA

Gene promoters with PARP1 binding motifs within 3kb upstream of the transcription start site identified from BLASTn search on the human RefSeq database. ^#^Gene promoter activity known to be regulated by PARP1. ^MBD4 interacts with MLH1 hence may also be involved in MMR [141,142].

## 5. Dysregulated PARP1 ADP-Ribosylation and Transcription Activities

**Figure 4:**
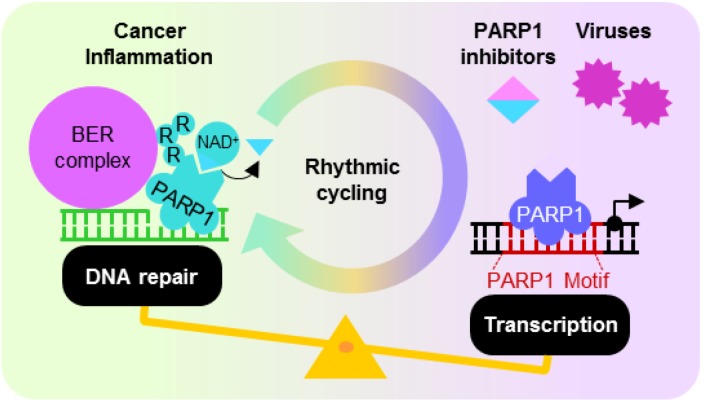
PARP1 DNA repair and motif-dependent transcription is intricately regulated and possibly temporally compartmentalized by the circadian rhythm. Disturbances to this may favor one function over the other, and arise from diseases such as cancer and inflammatory disorders, as well as the addition of small molecule inhibitors and utilization of PARP1 for viral replication.

Although PARP1 is heavily involved in DNA repair and transcriptional regulation, PARP1 activation at sites of DNA damage favors repair by shutting down transcription, recruiting polycomb and NuRD complexes that convert chromatin to its transcriptionally repressed state [147]. Conversely, as opposed to binding DNA strand breaks, sequence-specific motif binding suppresses the nuclear enzymatic activity of PARP1, reducing ADP-ribosylation on histone H1 and compromising cellular DNA repair [23]. However, the mechanism discriminating between intact binding motifs and damaged DNA remains unknown. Under physiological conditions, PARP1 ADP-ribosylation activity curiously follows the rhythmic circadian cycle [148]. The mechanism governing oscillating PARP1 enzymatic activity is not known, although in mice, autoregulatory loops with PARP1 acting as a transcriptional repressor at its own promoter may help to achieve this [149]. Rhythmic cycling of PARP1 ADP-ribosylation activity predicts for temporally compartmentalized DNA repair and transcription factor functions, where the efficacies of each is conversely related and differs throughout the day in a circadian rhythm-dependent manner (Figure 4). This intricate balance of PARP1 functions, however, may be perturbed in disease states such as cancer and inflammation, or by external agents such as small molecule PARP inhibitors and viruses, favoring one function over the other. The hepatitis B virus (HBV) provides a model to demonstrate this, whereby the utilization of motif–PARP1 interaction to drive viral replication suppresses PARP1 ADP-ribosylation activity hence compromises cellular DNA repair [23].

### 5.1. PARP1 Inhibition, Enzymatic Hyper-Activation and Disease

As the major contributor of ADP-ribosylation activities in the cell, PARP1 receives lots of attention for its enzymatic hyperactivation in various diseases. In particular, the reliance on it for initiating multiple DNA repair pathways, especially BER, HR and NHEJ, is exploited by cancer cells to safeguard against cell death induced by the accumulation of cytotoxic DNA lesions. As such, PARP1 expression is often elevated and its ADP-ribosylation activity increased in cancerous tissues [150,151], rendering it a good candidate for sensitizing cancer cells to the cytotoxic effects of DNA damaging agents [8,9,11,12,13,14,15,16,17]. Synthetic lethality may be achieved with PARP inhibitors designed to compete with NAD^+^ for the PARP1 catalytic site, and these small molecules have produced promising results in clinical trials for the treatment of several cancers. Further evidence for synthetic lethality was obtained in mice bearing BRCA1-deficient ovarian cancer cells, where life expectancy was significantly extended by delivery of nanoparticles containing siRNA targeting PARP1 [152]. PARP1 is also frequently implicated in inflammatory disorders such as sepsis, diabetes, myocardial infarction and stroke [8,9,11,12,15,16,109], as considerable by-stander DNA damage resulting from the generation of reactive oxygen species hyperactivates PARP1. Large amounts of NAD^+^ consumed in the process can also result in necrosis, aggravating the inflammatory condition. Under such circumstances, treatment with PARP1 inhibitors can provide symptomatic relief in animal models for such diseases [8,9,11,12,13,14,15,16,17,59,109], raising hope for clinical efficacy in the near future.

The ability of PARP1 to function without its enzymatic activity is often underappreciated. Indeed, many diseases involving PARP1 manifest independently of its ADP-ribosylation activity, and arise from dysregulated expression of molecules because of altered PARP1 affinity for its recognition motif in susceptibility genetic loci. Sequence-dependent PARP1 binding for SNP variants within promoter elements of the cytokine IL-10 [123], chemokine CCL2 [30], interferon α/β receptor 1 (IFNAR1) [27] and SMARCB1 [26], have all been associated with systemic lupus erythematosus (SLE), carotid atherosclerosis, chronic infection with HBV and acute lymphoblastic lymphoma (ALL), respectively. The effect of PARP1 inhibition on the outcome of such diseases, however, has not been evaluated. Importantly, because both PARP1 DNA repair and transcription factor functions may be implicated, the outcome of enzymatic inhibition in certain diseases need not be immediately conclusive. In diabetes, however, PARP inhibitors may be therapeutically beneficial as inhibiting ADP-ribosylation suppresses inflammation and concurrently enhances PARP1 transcription activator function at the Reg promoter for β-cell regeneration [29]. Interestingly, PARP1 inhibitors have gender-specific effects on animal disease models [153]. Whether this may also be true in the clinical setting remains to be seen.

### 5.2. Oncogenic Viruses—Inhibiting the PARP1 Enzyme to Enhance Viral Replication

The balance of PARP1 functions between enzyme-independent transcription and enzyme-dependent DNA repair is easily tipped by external stimuli. HBV is one external agent which deprives the infected host cell of PARP1 activity for its efficient replication whilst preventing its function in DNA repair, as the PARP1 binding motif “ACTTCAAA” carried within its genome is readily recognizable for transcriptional activation [23]. This may contribute to the oncogenic properties of HBV, especially in carriers of the virus with high HBV DNA loads [154,155,156]. By utilizing PARP1 to increase replication efficiency, the large amounts of viral DNA produced act as template to support further viral replication while concurrently inhibiting PARP1 ADP-ribosylation, reducing DNA repair capacity of the infected host cell. Accumulation of sublethal DNA lesions with prolonged infection [157] thus increases the risk of developing hepatocellular carcinoma (HCC). Several oncogenic viruses also possess high affinity PARP1 binding motifs in their genome (Table 3), suggesting that they may act on PARP1 in a similar manner to increase host risk for developing cancer. Viral DNA-PARP1 interaction has already been shown to be important for enhancing replication of the human T-cell leukemia virus (HTLV) [24] and the Kaposi’s sarcoma-associated virus (KSHV) [158,159]. Since these studies also show that PARP1 enzymatic inhibitors positively regulate viral replication, the use of small molecules targeting only the PARP1 catalytic domain may be contraindicated in many patients with such viral infections. Given the potential benefits of PARP inhibitors for the treatment of multiple diseases, it is perhaps worthwhile to invest in novel strategies that can overcome the pitfalls of aggravating viral replication and altered transcription factor function of current PARP inhibitors. 

**Table 3:** PARP1 binding motifs identified in oncogenic viral genomes.

Oncogenic virus	Gene or DNA element	Motif
Human herpesvirus 4 (EBV)	OriLyt replication origin	ACTTCAAA
Hepatitis B Virus (HBV)	Core promoter	ACTTCAAA
Human T-cell leukemia virus (HTLV)	Tax responsive element	ACGACAAC
Human herpesvirus 8 (KSHV)	ORF4 complement control protein	GCTACAAA
	Primase	ACGTCAAA
Merkel cell polyomavirus	VP3 capsid protein	ACTTCAAA

## 6. Conclusions

PARP1 plays important roles in both DNA repair and transcription, and the interplay of these processes in relation to cellular function and diseases states have not been well defined. As PARP1 binding motifs may be readily found in promoter elements of DNA repair genes, the expanding role of PARP1 in DNA repair need not be independent of transcription. Moreover, since PARP1 ADP-ribosylation is very important for DNA repair and transcription, yet the PAR-independent mechanism of transcriptional regulation through specific binding of PARP1 at its recognition motif also exists, rhythmic cycling of PARP1 enzyme activity suggests that these processes are unlikely to occur optimally together. The suppression of DNA repair by motif-dependent replication of oncogenic viruses illustrates this possibility. Thus, there is a need to better understand the effect of PARP1 inhibition in the therapeutic context and its effect on cellular transcription.
